# New Insight into Mercury Removal from Fish Meat Using a Single‐Component Solution Containing cysteine

**DOI:** 10.1002/gch2.202400161

**Published:** 2024-10-02

**Authors:** Przemysław Strachowski, Geeta Mandava, Johan Lundqvist, Romain Bordes, Mehdi Abdollahi

**Affiliations:** ^1^ Department of Life Sciences ‐ Food and Nutrition Science Chalmers University of Technology Gothenburg SE 412 96 Sweden; ^2^ Department of Biomedical Sciences and Veterinary Public Health ‐ Swedish University of Agricultural Sciences Uppsala SE 750 07 Sweden; ^3^ Department of Chemistry and Chemical Engineering ‐ Applied Chemistry Chalmers University of Technology Gothenburg SE 412 96 Sweden

**Keywords:** cysteine, detoxification, extraction, fish, food packaging, methylmercury

## Abstract

A novel approach for reducing mercury content in fish meat during post‐packaging storage is developed to extend the margin of their safe consumption. It involves employing a single‐component aqueous medium containing cysteine, as the active agent responsible for displacing mercury from fish proteins and its stabilization in the medium without the need for pH adjustments. The mercury removal efficiency depends on the cysteine concentration and its ratio to fish muscle. Using 1.2 wt% cysteine enables a reduction of mercury in canned Albacore tuna by 25–35%, depending on the fish product type and the exposure time of up to 2 weeks. The potential for the successful application of the developed method in active food packaging solutions is studied for the simultaneous or subsequent purification of the extraction solution through adsorption. Using thiolated silica could potentially enable the extraction process but it is shown that the presence of cysteine significantly hinders the adsorption.

## Graphical abstract

A novel method for reducing methylmercury in fish meat during storage is developed, using a single‐component aqueous cysteine solution. This approach achieves up to 35% mercury reduction in albacore tuna without pH adjustments. The study also explores mercury removal efficiency from solutions, highlighting cysteine's impact on adsorption processes and suggesting further research on adsorbent materials.

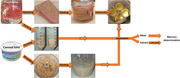

## 1 Introduction

While fish is widely recognized as nature's superfood providing many essential nutrients, there are inherent concerns related to its pollution with mercury. Moreover, the great majority of mercury bound in the fish muscles occurs in the most hazardous form of this element—namely methylmercury.^[^ ^1^, ^2^ ^]^ People's exposure to methylmercury primarily takes place through the consumption of contaminated fish and seafood. Upon entering the human body, it easily crosses the blood‐brain barrier and placenta, making it particularly hazardous to the developing fetus and young children.^[^ ^3^ ^]^ Chronic exposure to methylmercury can result in severe neurological and developmental impairments, including cognitive and motor function deficits. Given its persistent nature and ability to bioaccumulate, methylmercury remains a pressing global concern,^[^ ^4^ ^]^ necessitating stringent measures to reduce its release into the environment and mitigate the risks associated with its toxicity. World Health Organization (WHO) has placed mercury among the top chemicals of major public health concern.^[^ ^5^, ^6^ ^]^ Awareness of the problem exists both at the individual and governmental levels, resulting in visible improvements. However, the above‐mentioned bioaccumulative properties of mercury make the issue of fish pollution especially pressing. In this context, mistakes and neglect from the past will have long‐term consequences.

Some efforts are being made to reduce mercury emission and its spreading into the environment, however, currently there are no effective methods to reduce the risk related to mercury uptake via fish consumption. Development of solutions for mercury extraction from fish during packaging and shelf storage could be a promising and innovative alternative. This study introduces a new method of reducing mercury levels in fish through an improved extraction technique using a cysteine water solution as a packaging medium. The novelty of this approach is further enhanced by the adsorption of the extracted mercury onto thiolated silica. This method has the potential to enable the application of effective active packaging strategies, thereby increasing the safety of fish products for consumption.

The past studies^[^ ^7^ ^]^ clearly indicate that the application of extracting solutions could be a promising direction in this matter. However, in the face of a variety of fish species and methods of their processing, such a limited number of data does not allow for drawing well‐supported, systematical, and clear conclusions. There is no clear evidence of the influence of pH, time and solution volume and the form of the fish on the overall process efficiency. However, it is evident that the solution should comprise chelating compounds, preferably cysteine or EDTA. In addition, industrial implementation of these technologies has been barricaded due to their cost and possible side effects on product quality especially when they are used at low pH. Also using washing or dipping steps which are not common in processing the contaminated fish can result in loss of nutrients and water absorption.

Proteins in fish tissues, particularly sulfur‐containing amino acids within the protein structure, play a major role in trapping and accumulating mercury.^[^ ^8^ ^]^ The interaction between mercury and sulfur‐containing chemical groups (e.g., thiol) is well‐established and recognized as robust, as frequently documented in the literature.^[^ ^8^, ^9^, ^10^, ^11^, ^12^, ^13^ ^]^ Effective removal of mercury from fish tissues necessitates changing the affinity of mercury. To achieve this, a removing agent must directly access the trapped mercury. However, this step appears to be a challenge. Thermal treatment of fish meat, a common practice in the canning process, can lead to protein coagulation,^[^ ^14^ ^]^ thereby strengthening the mercury‐trapping effect. Consequently, it can be deduced that a potentially effective method for fish detoxification should satisfy the condition of partial penetration into the meat.

The objective of this study was to develop a two‐step method for removing mercury from fish products during packaging. This method involved initially treating the fish with a water solution (water‐based sauce) containing cysteine to extract mercury, followed by its adsorption onto powdered, thiolated silica and thiolated polymers. The proposed approach could potentially be implemented using adsorbent‐filled sachets placed within the container. We hypothesized that a simple and food‐friendly aqueous solution containing cysteine and silica‐filled sachet could effectively reduce the methylmercury level in fish meat during the storage of packaged products, such as fish canned or jarred in water‐based sauces. Thiolated silica was chosen due to strong evidence regarding the successful application of sulfur‐modified materials in mercury capturing from aqueous media.^[^ ^15^, ^16^, ^17^, ^18^, ^19^, ^20^ ^]^ In addition, the shelf storage period of the packed products is seen as an opportunity for promoting the extraction process and not requiring extra processing steps during production. Another alternative option could be simply discarding the water solution after opening the package and right before consumption.

The study proposes a potentially straightforward and applicable method for sequestering mercury from tuna meat. The developed approach involves utilizing a simple, single‐component water‐based solution without requiring pH adjustment, which can be effectively employed with food products. Cysteine, a well‐known and efficient mercury‐binding agent (which is explained in detail in the next paragraph), was selected as a food‐friendly component responsible for mercury capturing. Various parameters, including cysteine content, time duration, and fish form, were investigated to ascertain the most optimal conditions for mercury removal. The second part of the research included removing the collected mercury via the adsorption process using thiolated silica powder which was synthesized using a relatively simple method giving an efficient mercury adsorbent.

## 2 Experimental Section

### 2.1 Chemicals

European reference material of fish muscles ERM‐BB422 with a certified mercury content of 0.601 ± 0.030 mg kg^−1^ was purchased at the Joint Research Center website. Albacore Tuna canned in water and frozen Albacore tuna fillets (*Thunnus albacares*) were purchased from local stores. Cysteine, hydrochloric acid, citric acid, NaCl, EDTA (Merck) solutions were prepared with ultrapure water freshly produced with Milli‐Q Advantage A10 Water Purification System. The mesoporous silica (particle size of 3–5 µm, specific surface area 304 m^2^ g^−1^) microparticles were obtained from Nouryon (Bohus, Sweden). (3‐Mercaptopropyl)trimethoxysilane (3‐MPTMS), toluene, and sodium hydroxide were purchased from Merck. Methylmercury (II) chloride standard water solution with a concentration 1000 mg dm^−3^ (800 mg dm^−3^ Hg) was purchased from Alfa Aesar. Sulfur‐contained polymers polystyrene A SH and polystyrene AM SH were purchased from Rapp Polymere (Germany).

### 2.2 Development of Water‐Based Extraction Medium

**Figure 1:**
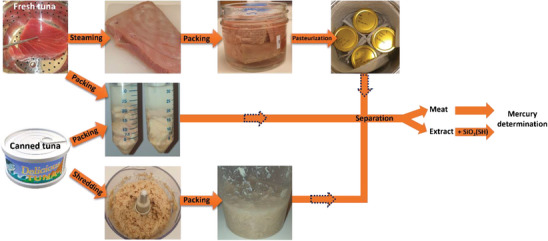
The overall scheme of the studies on detoxification of fresh, pasteurized, and canned tuna.

The overall scheme of the approach taken is presented in **Figure** **1**. The overarching goal of the experiments was to explore how different parameters of the process affect its efficacy in extracting mercury from fish meat. The study focused on examining the influence of both the solution composition and the form of the meat. The first experimental approach included systematical tests on the removal performance in relation to the cysteine concentration in the liquid phase. For this purpose, a series of cysteine/water solutions in the concentration range 0–5 wt% were prepared by dissolving the cysteine powder in MQ water. Then, the samples of fish including the whole pieces of fresh and canned tuna, minced canned tuna and steamed tuna were placed in the solution keeping 80 wt% of the solution and as prepared samples were stored at room temperature for 1 h. Mercury content (both in the solution and meat) allowed to present the results as the relationship between mercury removal efficacy versus cysteine concentration in the working solution.

The influence of the liquid/fish ratio in the system on the extraction performance was investigated by application of the solution composition found as the most promising based on the above‐described experiment. In this case, the samples of whole and minced pieces of canned tuna were placed in different volumes (10–80 wt%) of the extracting solution and the variations in the concentration of mercury were determined after 1 h storage.

The time dependence of the mercury removal in the time range between 1 to 12 h was also conducted. In this case, the fish pieces were placed in a plastic container and after adding the extracting solution (1.2 wt% of cysteine) the samples were stored at room temperature for a predefined time. The mercury concentration was determined by Hg analysis in the fish samples collected from the experiments. In addition, long‐time storage of the fish in the extracting solutions was conducted. This was to mimic the real storage conditions of the canned tuna and the canning‐like process was conducted in the laboratory by precooking, packing, and pasteurization the fresh fish purchased in the local fish store. In this case, the fresh filet (thickness of ca. 2–3 cm) was steamed for the time needed to heat its whole volume up to 70 °C (typically 7–9 min). The prepared fish meat was placed in 100 mL glass jar and filled with the extracting solution (1.2 wt% of cysteine) keeping the 60 wt% of the solution in each sample. Then, the samples were pasteurized to avoid spoilage and enable their long‐term storage. For this purpose, the jar, additionally sealed with Teflon tape was tightly closed, placed in water, and cooked at 90–95 °C for 25 min. The jars were then left overnight to cool down and stored at room temperature in darkness. After the predefined storage time (1–9 weeks), the samples were collected, the quality of the fish was assessed sensorily based on appearance and odor and finally, the mercury content was determined.

A description of the preliminary experiments including pH effects has been placed in the Supporting Information.

### 2.3 Preparation of Thiol‐Functionalized Silica Particles

Thiol‐functionalized silica particles were prepared using a two‐step process, alkaline activation and subsequent thiolation of the calcined material. Initially, the pristine silica was dried overnight at 105 °C, and then the desiccated material was placed in a plastic 100cm^3^ container. A 0.5 m NaOH water solution was introduced to the container, maintaining a silica mass to NaOH solution at a volume ratio of 1 g/5 cm^3^. The surface hydroxylation process was conducted at 50 °C for 60 min, using a water bath shaker. Following the completion of this step, the suspension was transferred to a centrifuge tube. Subsequently, the supernatant was decanted, and the powder underwent washing with water through a series of five cycles. The hydroxylated silica was then dried overnight at 105 °C and subjected to thiolation. In a round‐bottom flask, 2 g of the dry silica was dispersed in 50 cm^3^ of toluene, followed by the addition of 1.89 cm^3^ of 3‐MPTMS. The mixture was refluxed for 6 h. The resulting powder was then washed using toluene and ethanol. The as‐processed silica was dried overnight at 110 °C, and subsequently stored within a desiccator. The material is referred to as silica 6 h.

Additionally, a material called silica 24 h was synthesized with the methods described above, with the extension of refluxing time from 6 to 24 h. Silica 24 h turned out to be a hydrophobic material, thus it was subjected to slight, additional alkaline activation to increase its hydrophilicity. For this purpose, silica 24 h was suspended in 0.5 m solution of NaOH for 1 h at room temperature. Then, the material was separated, thoroughly rinsed with water to remove the residual NaOH, dried overnight at 105 °C and called silica 24 h (hydr).

### 2.4 Adsorption of Extracted Mercury with Thiolated Silica

The study on the adsorption was done by i) the adsorption of mercury from the extracts obtained in the previously described experiments and ii) the adsorption of mercury from the model systems obtained by preparing the 0.3 mg dm^−3^ solutions of mercury in the mixtures of stabilizing agents. The adsorption experiments were done by preparing the suspension of the aliquot of the adsorbent into the solution with various ratios between the solution volume and adsorbent mass. The as‐prepared samples were then shaken for 24 h using a rotary shaker. When the adsorption process was completed, the suspensions were centrifuged, and the supernatant was subjected to mercury content measurement using a direct mercury analyzer (DMA‐80 Direct Mercury Analysis System, Milestione, Italy) which allowed for quantitative evaluation of adsorption performance.

An aliquot of silica 6 h was placed in the solutions obtained from the extraction experiments (1 cm^3^ of extract : 20 mg of material) using different cysteine concentrations and minced canned tuna (look Figure 1) to investigate the influence of cysteine concentration on the adsorption performance. The second part here was to investigate the adsorption performance while it occurs in the different systems, namely using silica 24 h (hydr) and solutions obtained by mercury extraction from the raw fish by application of different cysteine concentrations.

The type of adsorbent and the influence of its amount on the adsorption were tested by using five different adsorbents including silica 6 h, silica 24 h, silica 24 h (hydr) and two sulfur‐containing polystyrene‐based polymers PS A SH and PS AM SH for the adsorption of mercury from the Hg‐containing solution obtained by storage of canned tuna in the 1.2 wt% solution of cysteine. For this purpose, the collected extract samples were exposed to different adsorbents with various solution volume : adsorbent mass ratio (mL:mg), namely 1:1.5; 1:5; 1:10; 1:15 and 1:20.

A series of adsorption experiments from the methylmercury/chelating agent mixtures was done. In these model systems, solutions with various compositions of cysteine, methylmercury, citric acid, EDTA, and NaCl were prepared using ultra‐pure water (compositions taken from the literature). This allowed for the reproduction of the conditions stabilizing the mercury in a water medium. Then, adsorption using silica 6 h was evaluated (1 cm^3^ of extract : 20 mg of material).

### 2.5 Mercury Analysis

Mercury analysis in the fish samples was conducted as follows: after a pre‐defined time, meat was separated from the solution by decantation in the case of fish pieces or by filtration through the cheesecloth when shredded fish was used. Then, the fish sample was rinsed with water for ca. 30 s and the meat separation process was repeated. The as‐obtained samples were subjected to freeze‐drying in order to achieve comparable, low moisture content which allows to obtain reliable results of remained mercury level after the process. This approach also allowed for a reliable comparison of the control samples that had no contact with any additional solutions. Generally, five types of fish samples were examined, namely, fish protein standard, minced and whole albacore tuna canned in water sauce, fresh tuna steak, and fresh tuna steak steamed and pasteurized in the laboratory. Mercury levels in fish meat and solutions were determined. Before the analysis, fish samples were freeze‐dried with a Labcono FreeZone tray dryer to achieve comparable moisture levels between different samples. In some cases, the fish was shredded using a kitchen blender.

The accuracy of the direct mercury analyzer (Milestone, DMA‐80) reading was assessed using certified fish protein standard ERM‐BB422 with a certified mercury content of 0.601 ± 0.030 mg kg^−1^. Five samples taken from the batch were analyzed and the average result was equal to 0.602 ± 0.031 mg kg^−1^.

## 3 Results and Discussion

### 3.1 Effect of Cysteine Concentration

**Figure 2:**
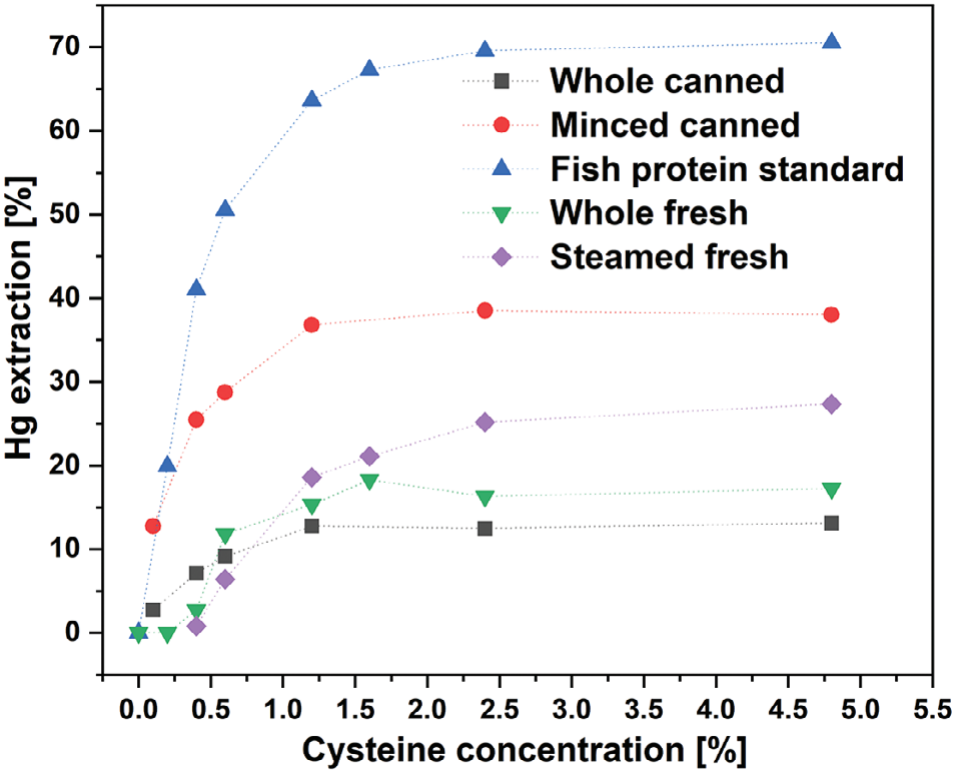
Mercury extraction from fish protein standard, the whole and minced canned Albacore tuna and whole fresh and steamed Albacore tuna with different cysteine concentrations (80% of solution, 1 h).

Cysteine concentration is a key parameter driving the mercury removal process due to its high affinity for mercury. The hypothesis, based on existing literature,^[^ ^7^, ^8^, ^9^, ^10^, ^11^, ^12^ ^]^ posits that an increase in cysteine content in the extracting solution will lead to a higher capacity of mercury scavenging from the fish tissue. The effect of cysteine concentration on mercury extraction from four kinds of fish form: i) fresh albacore tuna; ii) minced canned albacore tuna; iii) whole canned albacore tuna, iv) fresh albacore tuna steamed in the laboratory, v) the fish protein standard immersed in the solutions (80 wt% liquid content) for 1 h is presented in **Figure** **2**. The mercury extraction efficiency from different fish samples changed as follows: fish protein extract > minced canned fish > steamed fresh fish > fresh fish > whole canned fish and the percentage of the removed mercury was between 71 and 13%. The observed order was expected because of the availability of the tissue surface for the solution. The fish protein standard is a fine powder that forms a suspension in a water‐based medium, facilitating effective contact between the material and the solution. Moreover, when the fish material is completely dry, it efficiently absorbs water, enabling the extracting solution to fully penetrate the sample. Tests were furthermore done with real samples, first canned tuna in the original form of entire pieces (see Figure S1a, Supporting Information) and in a shredded form. The extraction efficiency was lower in comparison to the powdered proteins because of bigger tissue particles containing a natural level of moisture trapped in the structure. The limited availability of the fish sample for the solution, in the case of whole tuna pieces, resulted in approximately three times lower mercury reduction compared to the minced sample. Nevertheless, a 36% reduction of mercury level in the minced sample can be considered a promising result since it is a form of tuna commonly consumed (see Figure S1b, Supporting Information). The 3–4% higher mercury extraction from the fresh, unprocessed whole pieces of tuna (ca. 2 × 2× 2 cm) can be ascribed to a looser protein structure in that case (in comparison to the whole canned tuna). As mentioned above, thermal processing of the meat applied during the canning process may cause partial coagulation and lower mass transport in the meat.

From the curves of mercury extraction versus cysteine concentration, it can be concluded that in each type of fish sample, there is a threshold concentration of cysteine which above which no further mercury extraction is taking place. This most effective concentration of cysteine was found to be 1.2 wt% for both samples of canned fish, and 1.6 wt% in the case of fish protein standards and the fresh fish sample.

### 3.2 Effect of Time and Extracting Solution Content

**Figure 3:**
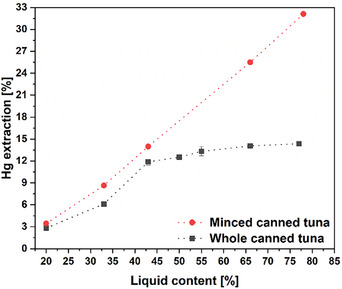
Mercury (Hg) extraction from whole and minced canned Albacore tuna using different volumes of the 1.2 wt% cysteine solution for 1 h.

**Figure 4:**
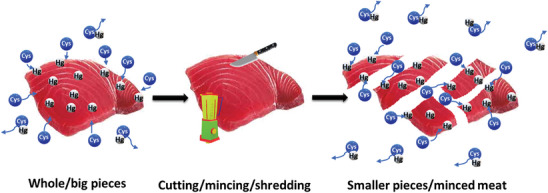
Schematical view of a possible mechanism for the effect of different‐sized fish pieces on mercury (Hg) extraction efficiency in the presence of cysteine (Cys).

Based on the results above, there is some upper limit of cysteine concentration allowing for mercury extraction with the highest possible efficiency. In this view, the duration and the working solution volume are naturally emerging questions. The solution content of 80 wt% used in our previous experiments was relatively high considering the real proportion in a can. The liquid content in water‐canned tuna is typically ca. 25–30 wt% based on the weight of the fish. **Figure** **3** presents the relation between mercury extraction efficiency and the volume of the extracting solution with a cysteine concentration of 1.2 wt% for the minced and whole canned tuna chosen as the representative samples here. Interestingly, two different behaviors were observed in the case of both fish samples. Reduction of the solution volume (keeping the fish mass constant) for the minced sample, has brought a linear decrease in the mercury extraction reaching approximately 8% for the level of 30% solution content. For the whole pieces of canned tuna, 45% solution content in the sample has been found as maximal and optimal for effective mercury removal. A decrease in the solution content in this case reduced extraction efficiency, but the increase, in turn, did not bring any noticeable changes. This that the availability of mercury‐contained protein in the fish tissue since meat particle size limits the availability of its external surfaces for the solution. A potential mechanism of extracting mercury involves the solution partially permeating the fish. Despite insufficient evidence about how deeply cysteine can infiltrate the muscle structure, the results strongly suggest that cysteine cannot diffuse throughout the entire fish specimen. This is why reducing the size of the pieces enhances the process. The extraction mechanism is schematically presented in **Figure** **4**.

**Figure 5:**
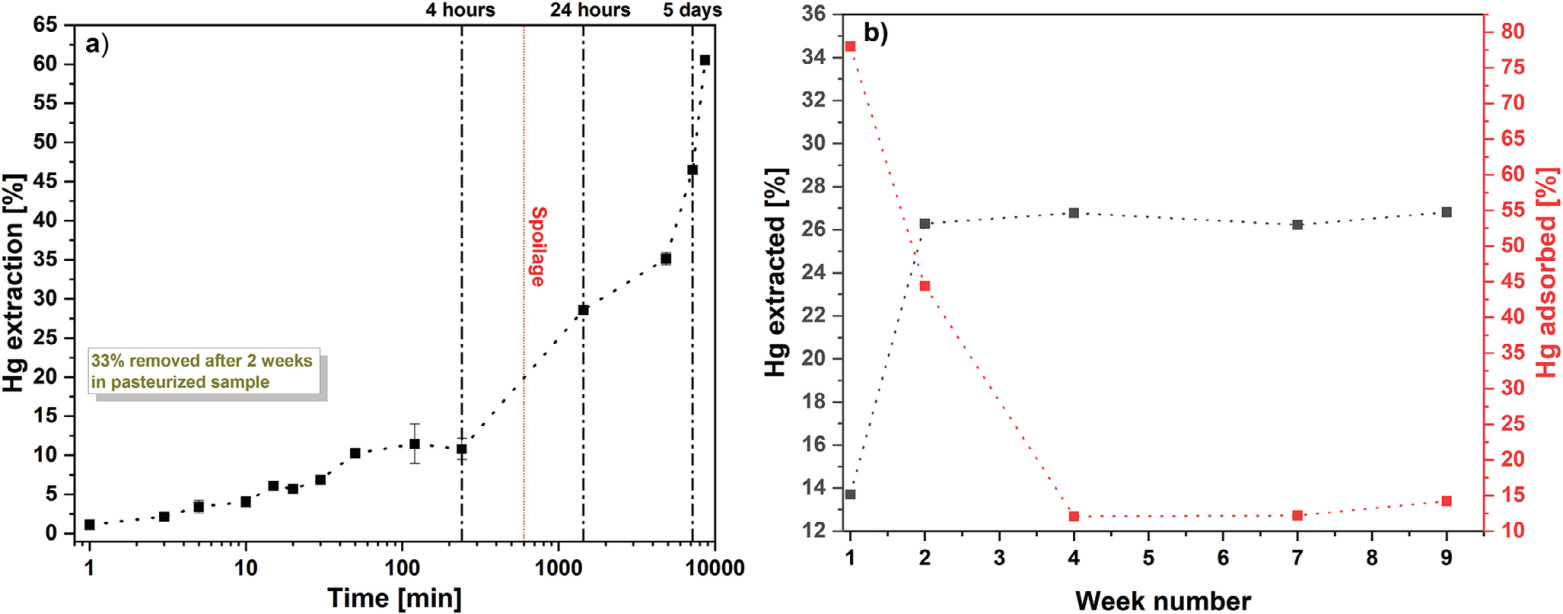
Time dependence of mercury extraction from a) whole canned tuna using 1.2 wt% cysteine solution, 80% of the solution, no pH adjustments (initial pH = 5.2); b) fresh tuna after precooking and pasteurization—black curve (1.2 wt%, no pH adjustment, 60% of solution), adsorption of Hg from the obtained extract (24 h, silica 6 h)—red curve.

The time dependence of the extraction process was also systematically investigated. **Figure** **5**a presents mercury extraction efficacy from whole pieces of canned tuna in 1.2 wt% of cysteine solution (80 wt% of solution content, logarithmic scale of x‐axis applied to clarify the picture) during 5 d of storage. Ca. 13% of mercury extraction yield was reached after 50 min, and no changes were observed up to 4 h of storage. Then a rapid growth in mercury extraction was noticed. However, the constant sensory control of the samples (smell and appearance) allowed to undoubtedly conclude that the fish spoilage began after 4 h. Spoilage of the fish causes various changes in its chemical structure, including protein degradation^[^ ^21^ ^]^ which can be responsible for the higher accessibility of mercury to the external environment, and this is precisely reflected in the course of the curve in Figure 5a. Moreover, as time progressed, the increase in extraction efficiency became more rapid, further reinforcing the conclusion about the substantial impact of mercury availability on its removal from the fish tissues. The higher the degree of spoilage, the higher protein degradation, and the weaker mercury trapping in the fish muscle structure.

Further extended experiments were carried out to gain a deeper understanding of the limitations of the developed approach to detoxifying fish. In this case, we steamed the fresh albacore tuna, packed it in jars containing 60 wt% of the 1.2 wt% cysteine solution and pasteurized it according to the method described in the methodology section. The results are presented in Figure 5b (black curve). The tests with no spoilage effect allowed to show that mercury is continuously extracted from the fish meat for up to 2 weeks, reaching 27%. After this time, the next 7 weeks, no further mercury extraction was observed. This is an important finding since in the case of preserved products storage time does not play a significant role and the timeframe of 2 weeks is rather not considered as an issue. Furthermore, it appeared that during the longer time of meat exposure to the extracting solution, cysteine had a chance to reach deeper trapped mercury. Another explanation of a delay of extraction is the gradual dissolution of sarcoplasmic proteins which is possible at the pH of the applied solutions.^[^ ^22^, ^23^, ^24^, ^25^, ^26^ ^]^

### 3.3 Adsorption of Mercury from the Solutions

Three types of thiolated silica powders were developed and tested as an adsorbent of mercury from the extract obtained during the process of fish detoxification using the cysteine solution. The capacity of methylmercury adsorption from the water solution onto the base material—silica 6 h reached the level of about 100 mg g^−1^ which can be considered as relatively high and enough for the purpose of the experiments (adsorption isotherm in Figure S2, Supporting Information). High effectivity of mercury adsorption from the pure water solution of methylmercury onto the silica 6 h was obtained, where the binding of mercury to thiol groups present on the surface of silica was carried out without the presence of interfering compounds.

**Figure 6:**
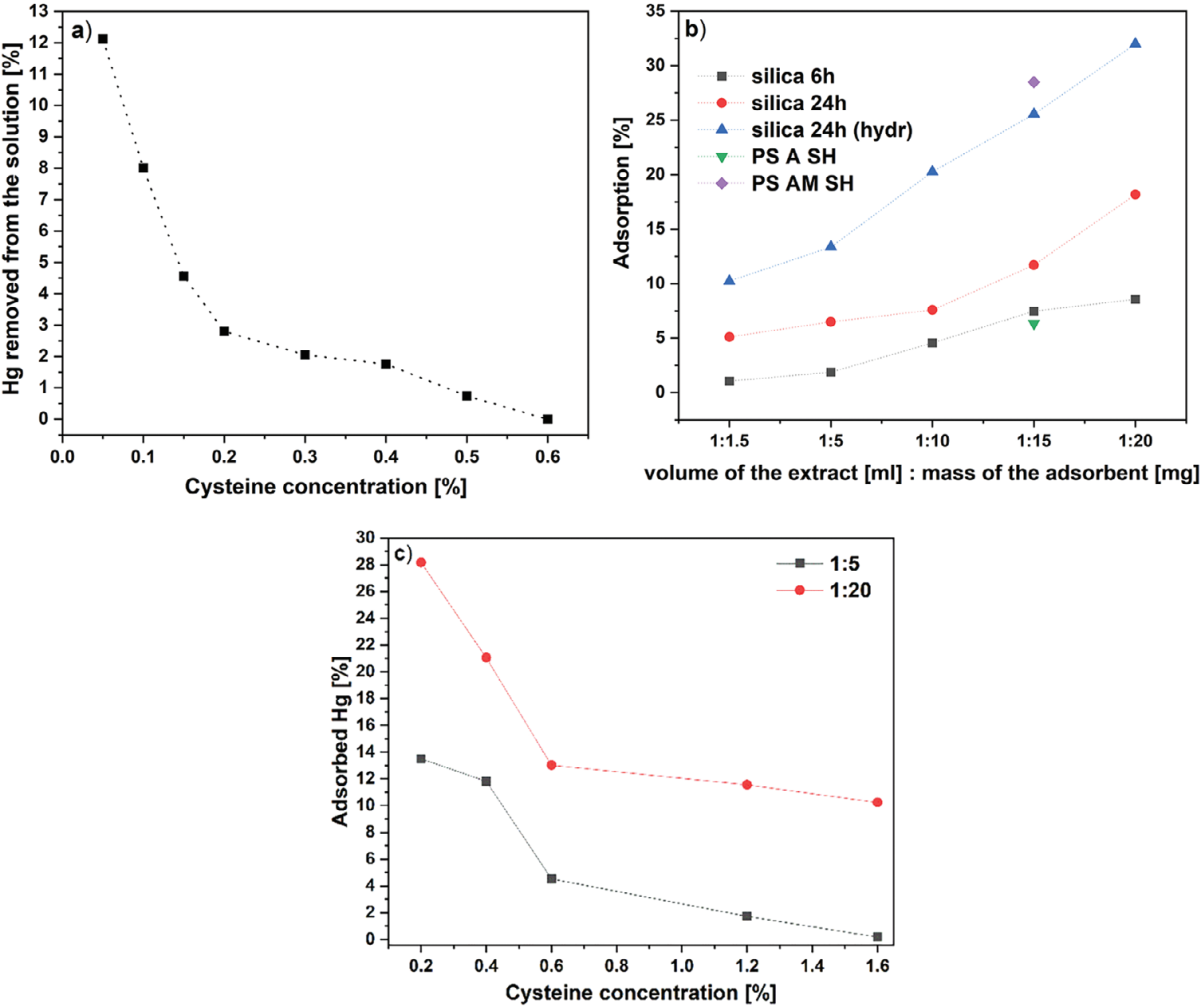
a) Adsorption of mercury from the extracts obtained from minced canned tuna with the solutions with different cysteine concentrations (24 h, silica 6 h). b) Adsorption of mercury from the extract obtained from a whole canned fish with the solution containing 1.2 wt% cysteine using different adsorbents with different volume: adsorbent ratios (initial mercury concentration 0.075 mg L^−1^). c) Adsorption of mercury from the extracts obtained from the raw fish (no cooking) with the solutions with different cysteine concentrations (silica 24 h (hydr)).

The first trial of mercury removal from the developed extracting solutions was conducted using the extract obtained from the detoxification of minced canned tuna with the solution containing different cysteine concentrations in the range between 0.5 and 1.2%. The results are presented in **Figure** **6**a as the relationship between the percentage of mercury removed from the solution and cysteine content. It is clearly seen that the presence of cysteine limited the adsorption efficiency and at the level of 0.6% totally prevented the adsorption process. That was an expected result since the interactions responsible for breaking the bonds between mercury and sulfur‐containing proteins are strong enough to keep mercury stable in the solution, thereby hindering the adsorption process. This finding has put the approach of simultaneous fish detoxification and removal of emerging mercury in the solution under a question mark since the same cysteine which is responsible for transferring the toxicant molecules from the solid state (fish tissue) to the liquid cannot easily transfer the toxicant from the liquid to another solid‐state material (silica 6 h). One of the possible straightforward scenarios to solve this issue was to increase the applied amount of the adsorbent. The results are plotted in Figure 6b and show that increasing the ratio between the mass of the material and the solution volume brings some positive effects. Mercury adsorption by silica 6 h raised from 0% to 7% which is still a very low level. The next step was to look for a more powerful adsorbent which was achieved by a longer silica thiolation process which resulted in obtaining silica 24 h and silica 24 h (hydr). In both cases, the higher amount of the powders resulted in a better adsorption efficiency and the highest obtained removal level was 33% (silica 24 (hydr)). Two single‐point adsorption experiments using commercially available sulfur‐containing polymers were also conducted. The results were comparable to those obtained using silica‐based adsorbents, whereas using thiolated silica is more economically justified.

Figure 6c presents the results for mercury adsorption using silica 24 h (hydr) from extracts containing different cysteine concentrations obtained from the raw tuna. The following trends i) the higher cysteine concentration the lower the mercury adsorption effectivity and ii) the higher the silica mass: extract volume ratio the higher the adsorption effectivity were still in place. However, a direct comparison of the results for silica 24 h (hydr) presented in Figure 6b for canned tuna and Figure 6c for fresh tuna allows to conclude that the adsorption effectivity is lower in the case of raw fish extract. This could be due the possible release of other compounds from the raw fish into the solution which further hinders the adsorption process through additional mercury stabilization or by directly affecting the adsorbent surface.

Further investigation of the possibilities of parallel or subsequent purification of the extracting solution included the preparation of the model systems based on data available in the literature.^[^ ^7^, ^27^ ^]^ In the case of these studies, the solutions were enriched with 0.3 mg L^−1^ methylmercury (0.3 mg L^−1^ of Hg from methylmercury) and then, the as‐prepared mixtures were subjected to the adsorption experiments using silica 6 h. The results are presented in **Table** **1**. Based on the results, it can be undoubtedly concluded that the presence of cysteine always hinders mercury adsorption. Other mercury chelators like EDTA and citric acid do not exhibit this behavior. However, some experiments in the laboratory of the authors of this paper indicated that the solutions containing EDTA, citric acid, and NaCl turned out to be ineffective in the case of mercury extraction from tuna, but the papers described the detoxification of mackerels.

**Table 1:** Mercury adsorption from the model solutions prepared according to the representative cases from the literature (Initial concentration 0.3 mg L^−1^).

Cys [g L^−1^]	EDTA [g L^−1^]	Citric acid [g L^−1^]	NaCl [g L^−1^]	% Hg removed (standard)	% of Hg extraction (ref.)	Refs.
6.3	0.137	–	2.5	8	78	[27]
12.5	0	–	5	10	70	
–	0.275	–	5	84	62	
–	–	0.4	0	100	65	[7]
–	–	31	5	89	74	
–	–	0.4	5	100	79	

## 4 Conclusions

A new simple method for the removal of mercury from tuna while soaked in canning water‐based mediums was successfully developed. Systematic studies proved that the application of 1.2 wt% cysteine solution enabled the reduction of mercury content in canned albacore tuna meat by 35% and its transfer to the canning medium at its natural pH during two weeks of storage. It was shown that increasing cysteine content in the extraction medium can increase mercury extraction efficiency but there is the clear upper limit for the cysteine content which is defined by the accessible level of mercury from the fish muscle. The efficiency of mercury extraction process from the fish using the cysteine solution depends on the size of the pieces of tuna (solid, chunk or mince) amount of the solution and time. Decreasing the size of the pieces of tuna in the can and increasing the solution ratio and storage time increased mercury extraction efficiency. The efficiency of the method was found independent from the additives such as organic acids or NaCl, and any pH adjustments.

The second part of the research assumed the possibility of simultaneous or subsequent removal of the extracted mercury from the solutions. Thiolated silica was found as an efficient adsorbent of mercury from aqueous media but the presence of cysteine, which is fully responsible for the mercury extraction from the fish, effectively prevented the adsorption. Increasing the thiolation amount and the mass of the adsorbent turned out to be a promising option here increasing the mercury adsorption to 30%. However, research on more powerful materials effectively capturing mercury even in the presence of cysteine should be a subject of further investigation.

Altogether, using the developed technology can be a promising approach for the reduction of consumers to mercury via canned tuna at least by 30% by simply separating the medium from canned tuna before consumption. Continuing the research on the development of more efficient adsorbents for incorporation in the can would be also a complementary route for adsorbing mercury from the medium and introducing novel active packing technologies. There is an undeniable need to develop safe and effective tools for reducing mercury levels in fish meat. These tools can complement the industry's efforts to minimize mercury release into the environment and offer new insights and ideas to enhance the safety of fish consumption.

## Conflict of Interest

The authors declare no conflict of interest.

## Supporting information



Supporting Information

## Data Availability

Data sharing is not applicable to this article as no new data were created or analyzed in this study.
