# Correction to “New Insight into Mercury Removal from Fish Meat Using a Single‐Component Solution Containing Cysteine”

**DOI:** 10.1002/gch2.202500170

**Published:** 2025-05-06

**Authors:** Przemysław Strachowski, Geeta Mandava, Johan Lundqvist, Romain Bordes, Mehdi Abdollahi

*Global Challenges* **2024**, *8*, 2400161

DOI: 10.1002/gch2.202400161

The error was due to the publication of material based on outdated “production data.” As a result, the published work did not include the section on effect‐based bioassays, which led to the omission of two full subsections describing the applied methods and the results, as well as the corresponding descriptions in the abstract and conclusion. The article should include:

**In abstract**: Additional last sentence “Based on the effect‐based in vitro screening, no relevant negative effects of the presented method on the safety of purified fish for humans are revealed.”

Two additional paragraphs:

2.6 Effect‐Based Screening Fish and Sauce

To evaluate the risk that the investigated process could contaminate the fish with potentially health‐hazardous compounds, an initial screening was performed with effect‐based methods in vitro. Fresh tuna was steamed, divided into portions, placed in 100 mL jars with 45 g of fish and 50 mL (the 1.2 wt.% cysteine solution), and then stored for two months. In parallel, two control samples were prepared: a) fish that was steamed and placed in jars but without the extracting solution and b) fish that was steamed and immediately frozen awaiting analysis (−80 °C). Following storage, the fish meat and the sauce were analyzed separately. Effect‐based methods in cultured mammalian cells were used to evaluate if there were compounds in the fish meat or the sauce that could activate a number of human health‐relevant toxicity endpoints. The fish meat was extracted using either a water‐based extraction or a methanol‐based extraction procedure. Extraction procedures are described in the Supporting Information. The sauce was added directly into the cell culture medium at an exposure concentration of 1%. A detailed methods description, including cell culture conditions, is presented in the Supporting Information. The applied in vitro assays are based on reporter gene technology, where the gene expression of a reporter protein is under the regulation of a DNA sequence that is responsive to the class of hazardous chemicals that are to be analyzed. Cell viability was monitored during all experiments, using the MTS assay, to ensure that analyses were conducted under non‐cytotoxic conditions. The samples were analyzed for estrogenic or androgenic effects, oxidative stress, and the potential to induce the aryl hydrocarbon receptor (AhR). A cell viability of >80% of the control cells was regarded as non‐cytotoxic.

3.4 Effect‐Based Evaluation of Potential Contamination by Developed Process

Fish meat that had been in contact with the cysteine solution was extracted and analyzed using in vitro bioassays to investigate if the developed process had contaminated the fish with any bioactive compounds. As controls, fish meat was stored in the same type of container but without the solution as was used, as well as fish meat that had been frozen awaiting analysis. All data is presented in Table S2 (Supporting Information). The fish meat extracted with a methanol‐based extraction method did not show any AhR activity, neither in the meat that had been in contact with the solution nor in both controls. The same was true for the extracts from the water‐based extraction.

The androgenic activity was observed in the meat that had been stored in the developed solution (both methanol‐based and water‐based extracts), but even higher androgenic activities were observed in the control meat that had been stored without the developed solution (water‐based extract) or the control meat that had been frozen (methanol‐based extract). Estrogenic effects were observed in the meat that had been stored in the developed solution (both methanol‐based and water‐based extracts). Estrogenic effects were also observed in both of the controls in the methanol‐based extracts, but not in any of the water‐based extracts. Oxidative stress was observed in all analyzed samples for both extraction methods.

Most of the activities observed in the meat that had been stored in the developed solution were also observed in either of the controls, at least to some degree, indicating that the developed solution was not causing the activity. It should, however, be noted that the estrogenic effect in the water‐based extract was only present in the extract from meat stored in the developed solution and not in the control. This warrants further investigations to clarify the cause of this effect. The sauce that had been stored with the fish was analyzed by direct addition in the cell culture medium (at 1%) without any extraction procedure. Neither estrogenicity, androgenicity, AhR activity nor oxidative stress response was observed in the sauce (data not shown).

**In conclusion**: Additional sentences at the end of the conclusion part: “The in vitro bioassays indicated that the developed cysteine solution did not introduce significant bioactive contaminants into the fish meat, as most observed activities were also present in the control samples, suggesting that the solution itself was not responsible for the detected effects.”

We apologize for this error.

