# Supplementary material for: New Insight into Mercury Removal from Fish Meat Using a Single‐Component Solution Containing cysteine
Source: Glob Chall. 2024 Oct 2;8(11):2400161. doi: 10.1002/gch2.202400161 (PMC11557507; doi:10.1002/gch2.202400161)
Supplement: Supplementary file 1 — Supporting Information [file GCH2-8-2400161-s001.docx]

Supplementary Data

**New insight into mercury removal from fish meat using a single-component solution containing cysteine**

*Przemysław Strachowski^*^, Geeta Mandava, Johan Lundqvist, Romain Bordes, Mehdi Abdollahi*


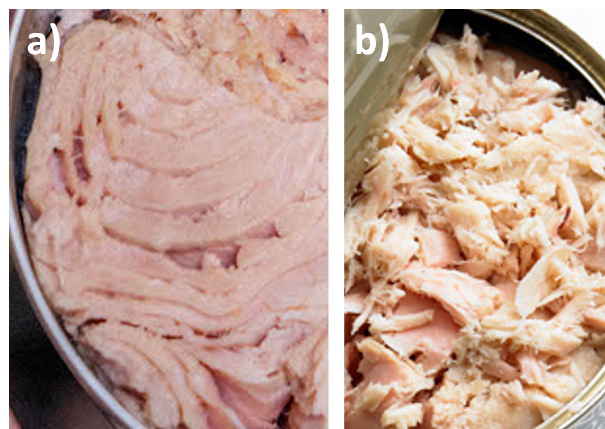

**Figure S1.** Commercially available (a) whole and (b) minced/shredded form of tuna canned in water.


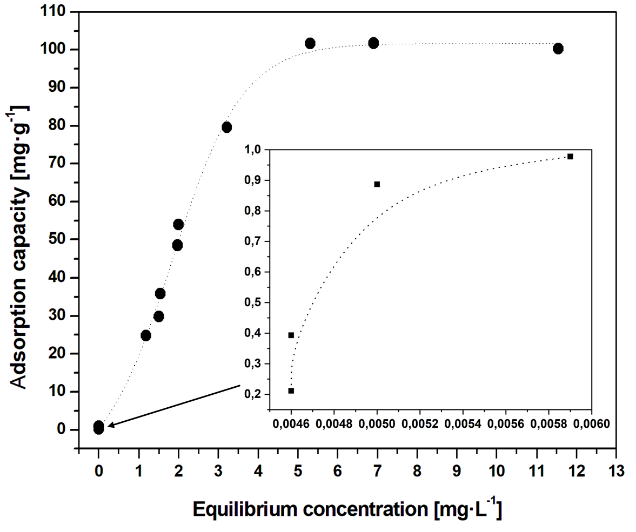


**Figure S2.** Isotherm of methylmercury adsorption onto silica 6h from water solution.

**Preliminary experiments**

The preliminary experiments aimed at extracting mercury from fish protein standards. as a model. and both from canned and fresh tuna using MQ water at pH 6.5 and water at pH 3.6. adjusted using diluted HCl. These experiments were conducted to establish a control point for subsequent studies. The form of the fish. the duration. pH and the amount of liquid medium used. showed no effect on mercury extraction efficiency. Mercury remained stable in the solid samples in the presence of water. This showed that simple water washing or soaking cannot remove mercury from fish and certain modifications to the solution are essential to achieve some satisfactory results in this field. Furthermore. it is important to highlight that no pH dependency for mercury removal efficiency in the pH range of 2.3-5.5 was observed in any of the conducted and subsequently described experiments. However. the available literature as summarized in the introduction still leaves the issue of pH dependence unclear. The absence of pH dependence found in this study can be regarded as a promising discovery since the elimination of pH adjustment streamlines the process. which is advantageous from an application standpoint.

The pH of acid-enriched solutions increased rapidly after 20-30s regardless of initial pH and the kind of acid. after its exposure to fish as shown in **Table** **S1**. Based on these findings. it can be concluded that pH may not play a significant role in mercury extraction. The initial low pH remained stable for only a few seconds. primarily due to the buffering effect of the fish tissue. which indicates that the mercury migration continues regardless of the pH changes.

**Table S1.** pH changes of the acid-containing solutions during exposure to the fish meat.

| Acid | Initial pH | Final pH |
| --- | --- | --- |
| Citric acid | 2.34 | 3.87 |
|  | 3.16 | 5.84 |
|  | 3.84 | 6.56 |
|  | 4.41 | 6.65 |
|  | 2.36 | 3.68 |
| Ascorbic acid | 3.07 | 4.89 |
|  | 3.32 | 5.74 |
|  | 3.88 | 5.65 |
|  | 4.6 | 6.73 |
|  | 2.93 | 4.4 |
| Hydrochloric acid | 2.98 | 6.15 |
|  | 3.62 | 6.26 |
|  | 4.16 | 6.63 |
|  | 4.58 | 6.65 |
|  | 2.71 | 5.98 |
| Acetic acid | 3.12 | 4.67 |
|  | 3.5 | 5.52 |
|  | 4.01 | 6.25 |
|  | 4.4 | 6.5 |
|  | 3.1 | 4.61 |

**Table S2.** The results of the effect-based evaluation of the technology of mercury removal from fish.

|  | **Fish stored in developed solution** | | **Fish stored without developed solution** | | **Fish frozen** | |
| --- | --- | --- | --- | --- | --- | --- |
|  | **Methanol-extract** | **Water-extract** | **Methanol-extract** | **Water-extract** | **Methanol-extract** | **Water-extract** |
| **Estrogenic activity, % of assay max^a^** | 136.7 | 51.3 | 28.7 | 10.2 | 42.4 | 14.6 |
| **Androgenic activity, % of assay max^a^** | 55.2 | 23.6 | 25.4 | 81.0 | 93.3 | 35.2 |
| **AhR activity, % of assay max^a^** | 13.0 | 9.5 | 8.6 | 12.5 | 5.1 | 16.5 |
| **Oxidative stress, fold change vs vehicle control^b^** | 5.7 | 7.3 | 3.4 | 3.0 | 2.8 | 5.3 |

^a^ >20% of assay max is defined as an active sample. ^b^ >1.5 fold change vs vehicle control is defined as an active sample.

**Section 1: Extraction-procedure for effect-based testing of fish meat**

Fish meat was extract wither with a methanol-based extraction method or a water-based extraction method. In the methanol-based extraction, 10 g of fish meat was minced in 23 mL methanol and left to settle. The supernatant was then collected and then diluted 1:100 in 0.001 M CaCl2 in MilliQ water. The solution was then filtered through a 0.45 µM filter before solid phase extraction (more details below). In the water-based extraction, 20 g of fish meat was minced in 200 mL 0.001 M CaCl2 in MilliQ water and left to settle. The supernatant was then collected and filtered through a 0.45 µM filter before solid phase extraction.

The solutions from both extraction methods then underwent solid phase extraction (SPE) with Oasis PRiME HLB 6 cc 200 mg columns (Waters), using an 8-channel automated SPE system (SPE-03 system, Promochrom Technologies). The SPE column was preconditioned with 5 mL 95% ethanol (5 mL/min) followed by 5 mL 95% methanol (5 mL/min) and 5 ml of 0.001 M CaCl2 in MilliQ water. The samples were then added with a flow rate of 20 mL/min, followed by air drying of the column by two times 5 mL of air. The column was then eluted with 2 times 5 mL 99% ethanol. The ethanol was evaporated by vacuum and exchanged for 150 µL of 99% ethanol. The concentrated samples were then diluted in cell culture medium 200 times.

**Section 2: Detailed description of the effect-based methods used, including cell culture conditions**

The stably transfected human breast adenocarcinoma cell line MCF-7/ARE c32 was used to measure oxidative stress corresponding to Nrf2 activity. The cells were obtained from Ximbio (London, UK). Cells were cultured in Dulbecco’s Modified Eagle Medium (DMEM GlutaMAX) with 4.5 g/L D-glucose completed with 10% fetal bovine serum (FBS), 1% of penicillin-streptomycin (100 units/mL penicillin, 100 µg/mL streptomycin) and 0.8 mg/mL Geneticin (G418) selective antibiotic (InvivoGen, USA). The experimental medium was DMEM GlutaMAX with 4.5 g/L D-glucose completed with 10% fetal bovine serum (FBS), 1% of penicillin-streptomycin.

AR EcoScreen GR-KO M1 cells, stably transfected CHO cells with GR knockout gene was used to assay human androgen receptor (AR) activity. The cells were obtained from Hiro Biotech via the Japanese Collection of Research Bioresources (JCRB), National Institutes of Biomedical Innovation, Health and Nutrition (Ibaraki city, Osaka, Japan). Cells were maintained in DMEM-F12 medium (Dulbecco's Modified Eagle Medium: Nutrient Mixture F-12, Sigma-Aldrich, Irvine, United Kingdom) supplemented with 10% FBS, 1% penicillin-streptomycin, 1% L-glutamine, 50 µg/mL Zeocin (Invitrogen, CA, USA) and 25 µg/mL Hygromycin B Gold (InvivoGen, CA, USA). The experimental medium contained DMEM-F12, 10% dextran charcoal treated FBS, 1% penicillin-streptomycin and 1% L-glutamine.

AhR transcriptional activation was evaluated in the mouse hepatoma cell line DR EcoScreen. The cells were obtained from Hiro Biotech via the Japanese Collection of Research Bioresources (JCRB), National Institutes of Biomedical Innovation, Health and Nutrition (Ibaraki city, Osaka, Japan). Culture medium consisted of α-Minimum Essential Media (α-MEM), 5% FBS, 1% penicillin-streptomycin, 1% L glutamine and 150 µg/mL Hygromycin B Gold (InvivoGen, USA). The experimental medium consisted of α-MEM, 5% FBS and, 1% penicillin-streptomycin.

Estrogen Receptor Luciferase Reporter T47D Stable Cell Line was obtained from Signosis Inc (Santa Clara, CA). The cells were routinely cultured in medium consisting of Roswell Park Memorial Institute (RPMI) 1640 media with L-glutamine complemented with 10% FBS, 1% penicillin-streptomycin, and 75µg/ml G418. During the experiments, the cells were cultured in an estrogen-free medium consisting of DMEM with 4.5 g/L glucose, sodium pyruvate, without phenol red and complemented with 5% dextran/charcoal treated FBS, 2% L-glutamine, and 1% penicillin-streptomycin.

All mentioned cell culture supplements and media were purchased from Gibco, Thermo Fisher Scientific, unless otherwise stated.

Cells were maintained in an incubator at 37˚C in 5% CO2. Medium was changed every second to third day. Trypsin-EDTA (Gibco, Thermo Fisher Scientific) was used for passaging of cells.

Cells for reporter gene assays were seeded in 384 well white-walled plates with clear bottom (number of cells per well; 4000 cells for DR-EcoScreen, 4000 cells for AR-EcoScreen, 20000 cells for T47D, and 5200 cells for AREc32). The cells were then incubated for 24 h before they were exposed. Following exposure, the cells were incubated for 24 h and then lysed and the luminescence was measured using Luciferase Assay System reagents (Promega). The luminescence was measured using a Spark Multimode Microplate Reader (TECAN, Austria, GmbH) with an automatic syringe injector for addition of the luciferase substrate. Cell viability was assayed in parallel, using the MTS assay.
